# Supplementary material for: Sample composition alters associations between age and brain structure
Source: Nat Commun. 2017 Oct 12;8:874. doi: 10.1038/s41467-017-00908-7 (PMC5638928; doi:10.1038/s41467-017-00908-7)
Supplement: Supplementary file 1 — Supplementary Information [file 41467_2017_908_MOESM1_ESM.pdf]

Supplementary Information

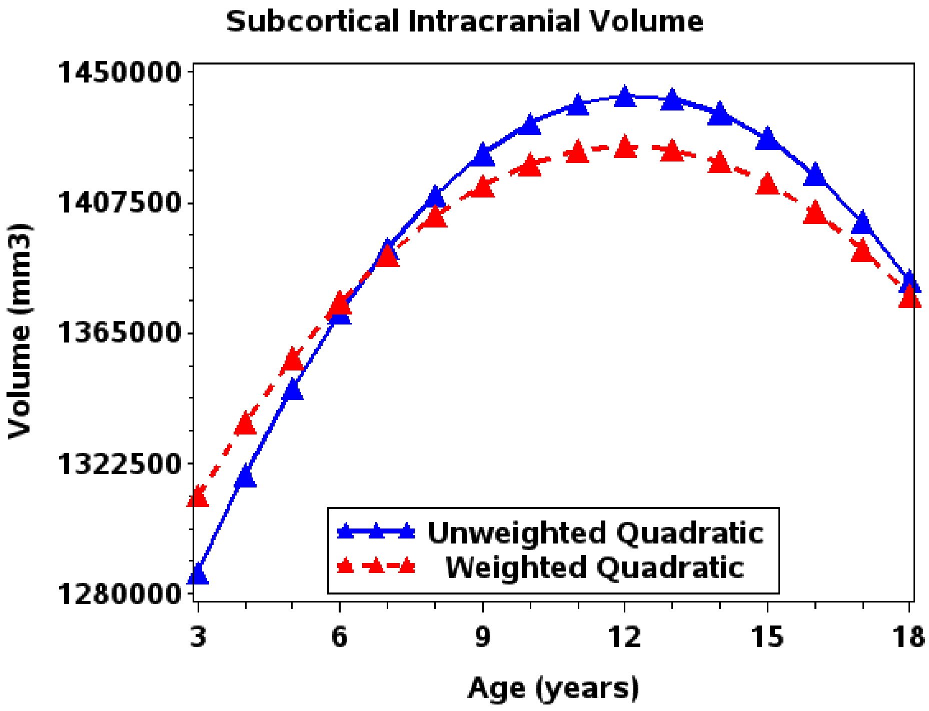

Supplementary Figure 1: Age-related variation in intracranial volume in the unweighted and weighted samples. Using the best-fitting regression models described in Supplementary Table 6, we calculated the predicted values by age for subcortical intracranial volume in the unweighted (blue lines) and weighted (red lines) samples.

Supplementary Table 1. Brain regions included in lobe specific analyses. The individual cortical surface area and thickness regions included in each lobe specific analysis (frontal, parietal, temporal and occipital) as identified in the Desikan-Killiany atlas.

Lobe	Individual Regions Included
Frontal Lobe	Superior Frontal<br>Rostral and Caudal Middle Frontal<br>Pars Opercularis<br>Pars Triangularis<br>Pars Orbitalis<br>Lateral and Medial Orbitofrontal<br>Precentral<br>Paracentral<br>Frontal Pole<br>Rostral Anterior<br>Caudal Anterior
Parietal Lobe	Superior Parietal<br>Inferior Parietal<br>Supramarginal<br>Postcentral<br>Precuneus<br>Posterior<br>Isthmus
Temporal Lobe	Superior, Middle, and Inferior Temporal<br>Banks of the Superior Temporal Sulcus<br>Fusiform<br>Transverse Temporal<br>Entorhinal<br>Temporal Pole<br>Parahippocampal
Occipital Lobe	Lateral Occipital<br>Lingual<br>Cuneus<br>Pericalcarine

Supplementary Table 2. Best fitting models for hemispheric, global measures of brain structure. Beta estimates, standard errors (SE), and AIC fit statistics for linear, quadratic, and cubic models of age for left and right hemispheric cortical volume, mean cortical thickness, and cortical surface area in both the unweighted and weighted PING data. The AIC of the best fitting model for each outcome is in bold.

			Unweighted PING Data		Weighted PING Data	
			**Beta (SE)**	**AIC**	**Beta (SE)**	**AIC**
Left Total Cortical Volume	Linear	Age	-3018.1 (137.81)	22772.64	-3230.21 (141.95)	23689.76
	Quadratic	Age	-2913.68 (140.63)	**22763.68**	-3183.88 (141.84)	23680.61
		Age^2	-114.28 (34.42)		-118.32 (35.34)	
	Cubic	Age	-3161.03 (311.95)	22764.89	-4005.11 (324.3)	**23674.73**
		Age^2	-120.6 (35.13)		-126.81 (35.33)	
		Age^3	7.54 (8.49)		24.73 (8.79)	
Right Total Cortical Volume	Linear	Age	-3033.88 (139.1)	22791.57	-3291.08 (144.65)	23728.12
	Quadratic	Age	-2919.12 (141.8)	**22780.55**	-3236.18 (144.27)	23715.02
		Age^2	-125.59 (34.71)		-140.21 (35.94)	
	Cubic	Age	-3198.6 (314.52)	22781.56	-4012.32 (330.02)	**23710.21**
		Age^2	-132.74 (35.42)		-148.22 (35.95)	
		Age^3	8.53 (8.56)		23.37 (8.95)	
Left Total Thickness	Linear	Age	-0.029 (8.0E-4)	-1699.38	-0.03 (8.0E-4)	**-782.32**
	Quadratic	Age	-0.029 (8.0E-4)	-1702.62	-0.03 (8.0E-4)	-780.44
		Age^2	0.001 (2.0E-4)		0 (2.0E-4)	
	Cubic	Age	-0.025 (1.8E-3)	**-1705.80**	-0.026 (1.9E-3)	-783.10
		Age^2	0.001 (2.0E-4)		0 (2.0E-4)	
		Age^3	0 (0.0E+0)		0 (1.0E-4)	
Right Total Thickness	Linear	Age	-0.029 (8.0E-4)	-1634.83	-0.031 (8.0E-4)	-741.40
	Quadratic	Age	-0.03 (8.0E-4)	-1640.64	-0.031 (8.0E-4)	-739.60
		Age^2	0.001 (2.0E-4)		0 (2.0E-4)	
	Cubic	Age	-0.026 (1.9E-3)	**-1643.47**	-0.026 (1.9E-3)	**-743.97**
		Age^2	0.001 (2.0E-4)		0 (2.0E-4)	
		Age^3	0 (1.0E-4)		0 (1.0E-4)	
Left Total Cortical Surface Area	Linear	Age	303.19 (66.01)	21287.43	185.84 (66.39)	22148.75
	Quadratic	Age	405.6 (65.66)	**21234.33**	220.06 (65.6)	22118.08
		Age^2	-120.61 (16.03)		-93.77 (16.28)	
	Cubic	Age	134.14 (146.54)	21232.05	-305.06 (149.82)	**22105.05**
		Age^2	-127.5 (16.34)		-98.97 (16.21)	
		Age^3	8.28 (4)		15.8 (4.06)	
Right Total Cortical Surface Area	Linear	Age	296.91 (67.07)	21319.90	189.39 (67.45)	22180.92
	Quadratic	Age	400.53 (66.74)	**21267.28**	223.5 (66.69)	22151.49
		Age^2	-122.03 (16.29)		-93.48 (16.55)	
	Cubic	Age	136.8 (148.96)	21265.37	-303.93 (152.33)	**22138.82**
		Age^2	-128.73 (16.61)		-98.7 (16.48)	
		Age^3	8.04 (4.06)		15.87 (4.13)	

Supplementary Table 3. Best fitting models for lobe specific cortical thickness. Beta estimates, standard errors (SE), and AIC fit statistics for linear, quadratic, and cubic models of age for left and right frontal, occipital, temporal, and parietal lobe thickness in both the unweighted and weighted PING data. The AIC of the best fitting model for each outcome is in bold.

			**Unweighted PING Data**		**Weighted PING Data**	
			**Beta (SE)**	**AIC**	**Beta (SE)**	**AIC**
Left Frontal Lobe Thickness	Linear	Age	-0.034 (1.0E-3)	-1323.84	-0.035 (1.0E-3)	-358.81
	Quadratic	Age	-0.035 (1.0E-3)	-1340.85	-0.035 (1.0E-3)	-368.81
		Age^2	0.001 (2.0E-4)		0.001 (3.0E-4)	
	Cubic	Age	-0.029 (2.2E-3)	**-1347.18**	-0.029 (2.3E-3)	**-376.14**
		Age^2	0.001 (2.0E-4)		0.001 (3.0E-4)	
		Age^3	0 (1.0E-4)		0 (1.0E-4)	
Right Frontal Lobe Thickness	Linear	Age	-0.033 (1.0E-3)	-1176.03	-0.035 (1.0E-3)	-316.74
	Quadratic	Age	-0.034 (1.0E-3)	**-1188.79**	-0.035 (1.0E-3)	**-320.04**
		Age^2	0.001 (3.0E-4)		0.001 (3.0E-4)	
	Cubic	Age	-0.031 (2.3E-3)	-1189.32	-0.032 (2.4E-3)	-320.38
		Age^2	0.001 (3.0E-4)		0.001 (3.0E-4)	
		Age^3	0 (1.0E-4)		0 (1.0E-4)	
Left Occipital Lobe Thickness	Linear	Age	-0.115 (4.0E-3)	1585.00	-0.116 (4.1E-3)	2481.75
	Quadratic	Age	-0.12 (4.0E-3)	1549.63	-0.118 (4.1E-3)	**2458.34**
		Age^2	0.006 (1.0E-3)		0.005 (1.0E-3)	
	Cubic	Age	-0.099 (9.0E-3)	**1545.08**	-0.115 (9.4E-3)	2460.22
		Age^2	0.007 (1.0E-3)		0.005 (1.0E-3)	
		Age^3	-0.001 (2.0E-4)		0 (3.0E-4)	
Right Occipital Lobe Thickness	Linear	Age	-0.119 (3.7E-3)	1437.02	-0.128 (3.8E-3)	2302.28
	Quadratic	Age	-0.125 (3.7E-3)	1379.06	-0.13 (3.7E-3)	**2279.90**
		Age^2	0.007 (9.0E-4)		0.005 (9.0E-4)	
	Cubic	Age	-0.1 (8.3E-3)	**1369.26**	-0.117 (8.6E-3)	2278.95
		Age^2	0.008 (9.0E-4)		0.005 (9.0E-4)	
		Age^3	-0.001 (2.0E-4)		0 (2.0E-4)	
Left Temporal Lobe Thickness	Linear	Age	-0.02 (1.1E-3)	**-972.49**	-0.019 (1.1E-3)	**-125.05**
	Quadratic	Age	-0.02 (1.2E-3)	-970.84	-0.019 (1.1E-3)	-123.42
		Age^2	0 (3.0E-4)		0 (3.0E-4)	
	Cubic	Age	0.02 (2.6E-3)	-968.92	-0.019 (2.6E-3)	-121.42
		Age^2	0 (3.0E-4)		0 (3.0E-4)	
		Age^3	0 (1.0E-4)		0 (1.0E-4)	
Right Temporal Lobe Thickness	Linear	Age	-0.018 (1.1E-3)	**-1103.58**	-0.019 (1.1E-3)	**-208.97**
	Quadratic	Age	-0.018 (1.1E-3)	-1102.35	-0.019 (1.1E-3)	-211.37
		Age^2	0 (3.0E-4)		-0.001 (3.0E-4)	
	Cubic	Age	-0.015 (2.4E-3)	-1103.20	-0.016 (2.5E-3)	-211.37
		Age^2	0 (3.0E-4)		-0.001 (3.0E-4)	
		Age^3	0 (1.0E-4)		0 (1.0E-4)	
Left Parietal Lobe Thickness	Linear	Age	-0.031 (8.0E-4)	**-1629.68**	-0.033 (9.0E-4)	**-680.48**
	Quadratic	Age	-0.031 (8.0E-4)	-1630.29	-0.033 (9.0E-4)	-679.67
		Age^2	0 (2.0E-4)		0 (2.0E-4)	
	Cubic	Age	-0.028 (1.9E-3)	-1630.89	-0.032 (2.0E-3)	-677.78
		Age^2	0 (2.0E-4)		0 (2.0E-4)	
		Age^3	0 (1.0E-4)		0 (1.0E-4)	
Right Parietal Lobe Thickness	Linear	Age	-0.031 (9.0E-4)	-1562.38	-0.033 (9.0E-4)	-646.34
	Quadratic	Age	-0.031 (9.0E-4)	**-1565.33**	-0.033 (9.0E-4)	-644.47
		Age^2	0.001 (2.0E-4)		0 (2.0E-4)	
	Cubic	Age	-0.029 (1.9E-3)	-1565.31	-0.028 (2.0E-3)	**-649.14**
		Age^2	0.001 (2.0E-4)		0 (2.0E-4)	
		Age^3	0 (1.0E-4)		0 (1.0E-4)	

Supplementary Table 4. Best fitting models for lobe specific cortical surface area. Beta estimates, standard errors (SE), and AIC fit statistics for linear, quadratic, and cubic models of age for left and right frontal, occipital, temporal, and parietal lobe surface area in both the unweighted and weighted PING data. The AIC of the best fitting model for each outcome is in bold.

			**Unweighted PING Data**		**Weighted PING Data**	
			**Beta (SE)**	**AIC**	**Beta (SE)**	**AIC**
Left Frontal Lobe Surface Area	Linear	Age	162.8 (25.96)	19392.66	147 (26.85)	20311.13
	Quadratic	Age	203.18 (25.82)	**19339.26**	159.3 (26.62)	20287.38
		Age^2	-47.55 (6.3)		-33.73 (6.61)	
	Cubic	Age	112.9 (57.65)	19338.20	-4.54 (60.99)	**20280.52**
		Age^2	-49.84 (6.43)		-35.35 (6.6)	
		Age^3	2.75 (1.57)		4.93 (1.65)	
Right Frontal Lobe Surface Area	Linear	Age	155.15 (26.37)	19424.74	137.47 (27.27)	20342.19
	Quadratic	Age	195.27 (26.26)	**19373.79**	149.82 (27.04)	20319.06
		Age^2	-47.25 (6.41)		-33.84 (6.71)	
	Cubic	Age	119.49 (58.67)	19373.71	6.87 (62.02)	**20314.53**
		Age^2	-49.18 (6.54)		-35.26 (6.71)	
		Age^3	2.31 (1.6)		4.3 (1.68)	
Left Occipital Lobe Surface Area	Linear	Age	47.67 (10.41)	17538.00	21.04 (11.04)	18507.05
	Quadratic	Age	56.61 (10.55)	**17523.43**	24.48 (11.02)	18497.25
		Age^2	-10.53 (2.58)		-9.42 (2.73)	
	Cubic	Age	14.71 (23.56)	17521.49	-69.11 (25.15)	**18482.31**
		Age^2	-11.59 (2.63)		-10.35 (2.72)	
		Age^3	1.28 (0.64)		2.82 (0.68)	
Right Occipital Lobe Surface Area	Linear	Age	35.89 (10.84)	17620.73	14.27 (11.2)	18536.86
	Quadratic	Age	46.52 (10.97)	**17601.11**	17.83 (11.18)	18526.59
		Age^2	-12.51 (2.68)		-9.75 (2.77)	
	Cubic	Age	4.91 (24.48)	17599.51	-80.89 (25.5)	**18510.28**
		Age^2	-13.57 (2.73)		-10.73 (2.76)	
		Age^3	1.27 (0.67)		2.97 (0.69)	
Left Temporal Lobe Surface Area	Linear	Age	81.85 (15.03)	18283.06	52.59 (14.62)	19076.56
	Quadratic	Age	106.67 (14.89)	**18222.37**	62.05 (14.31)	19026.50
		Age^2	-29.23 (3.63)		-25.94 (3.55)	
	Cubic	Age	60.43 (33.26)	18221.96	-38.81 (32.73)	**19016.86**
		Age^2	-30.4 (3.71)		-26.94 (3.54)	
		Age^3	1.41 (0.91)		3.03 (0.89)	
Right Temporal Lobe Surface Area	Linear	Age	91.52 (14.26)	18176.85	73.07 (13.61)	18931.83
	Quadratic	Age	113.26 (14.2)	**18125.69**	81.47 (13.35)	18886.64
		Age^2	-25.6 (3.47)		-23.03 (3.31)	
	Cubic	Age	76.09 (31.73)	18125.97	-8.24 (30.56)	**18878.07**
		Age^2	-26.55 (3.54)		-23.91 (3.31)	
		Age^3	1.13 (0.87)		2.7 (0.83)	
Left Parietal Lobe Surface Area	Linear	Age	10.87 (20.88)	18950.88	-34.78 (20.27)	19739.81
	Quadratic	Age	39.15 (20.91)	18911.17	-25.78 (20.11)	19717.62
		Age^2	-33.3 (5.1)		-24.68 (4.99)	
	Cubic	Age	-53.9 (46.64)	**18908.21**	-192.61 (45.9)	**19703.46**
		Age^2	-35.66 (5.2)		-26.33 (4.97)	
		Age^3	2.84 (1.27)		5.02 (1.24)	
Right Parietal Lobe Surface Area	Linear	Age	14.36 (21.84)	19042.56	-35.42 (21.48)	19858.34
	Quadratic	Age	45.49 (21.82)	18998.27	-25.62 (21.3)	19834.83
		Age^2	-36.66 (5.33)		-26.87 (5.29)	
	Cubic	Age	-63.68 (48.66)	**18993.99**	-221.68 (48.53)	**19816.90**
		Age^2	-39.43 (5.42)		-28.8 (5.25)	
		Age^3	3.33 (1.33)		5.9 (1.32)	

Supplementary Table 5. Best fitting models for subcortical regions. Beta estimates, standard errors (SE), and AIC fit statistics for linear, quadratic, and cubic models of age for total bilateral amygdala, hippocampal and basal ganglia volume in both the unweighted and weighted PING data. The AIC of the best fitting model for each outcome is in bold.

			**Unweighted PING Data**		**Weighted PING Data**	
			**Beta (SE)**	**AIC**	**Beta (SE)**	**AIC**
Amygdala Volume (bilateral)	Linear	Age	40.28 (4.41)	15783.63	33.38 (4.7)	16772.94
	Quadratic	Age	45.77 (4.45)	**15755.70**	36 (4.65)	16742.13
		Age^2	-6.01 (1.09)		-6.69 (1.16)	
	Cubic	Age	30.15 (9.87)	15754.57	-4.23 (10.58)	**16726.44**
		Age^2	-6.41 (1.11)		-7.1 (1.15)	
		Age^3	0.48 (0.27)		1.21 (0.29)	
Basal Ganglia Volume (bilateral)	Linear	Age	121.6 (17.05)	18529.90	99.86 (17.09)	19391.93
	Quadratic	Age	143.18 (17.22)	**18500.98**	111.4 (16.76)	19345.27
		Age^2	-23.62 (4.22)		-29.48 (4.18)	
	Cubic	Age	129.59 (38.22)	18502.82	-49.11 (38.06)	**19325.55**
		Age^2	-23.97 (4.3)		-31.14 (4.15)	
		Age^3	0.41 (1.04)		4.83 (1.03)	
Hippocampus Volume (bilateral)	Linear	Age	39.62 (4.89)	15994.46	33.7 (5.07)	16924.35
	Quadratic	Age	43.75 (4.98)	**15982.86**	34.76 (5.08)	**16921.81**
		Age^2	-4.51 (1.22)		-2.7 (1.27)	
	Cubic	Age	31.22 (11.05)	15983.25	18.79 (11.65)	16921.49
		Age^2	-4.83 (1.24)		-2.86 (1.27)	
		Age^3	0.38 (0.3)		0.48 (0.32)	

Supplementary Table 6. Best fitting model for intracranial subcortical volume. Beta estimates, standard errors (SE), and AIC fit statistics for linear, quadratic, and cubic models of age for subcortical intracranial volume in both the unweighted and weighted PING data. The AIC of the best fitting model for each outcome is in bold.

**Intracranial Volume**						
			**Unweighted PING Data**		**Weighted PING Data**	
			**Beta (SE)**	**AIC**	**Beta (SE)**	**AIC**
Intracranial Volume	Linear	Age	4991.99 (1491.81)	27616.95	2871.19 (1538.72)	28529.25
	Quadratic	Age<br>Age^2	6537.27 (1506.86)<br>-1819.79 (367.79)	**27594.76**	3376.92 (1535.08)<br>-1386.07 (380.85)	**28518.09**
	Cubic	Age<br>Age^2<br>Age^3	5783.04 (3369.7)<br>-1838.95 (375.66)<br>22.99 (91.89)	27596.70	167.39 (3529.96)<br>-1417.8 (381.95)<br>96.56 (95.64)	28519.07
