# Supplementary material for: Sample composition alters associations between age and brain structure
Source: Nat Commun. 2017 Oct 12;8:874. doi: 10.1038/s41467-017-00908-7 (PMC5638928; doi:10.1038/s41467-017-00908-7)
Supplement: Supplementary file 3 — Description of Additional Supplementary Files [file 41467_2017_908_MOESM3_ESM.pdf]

File name: Supplementary Data 1
Description: Statistical code used to generate the results summarized in this manuscript entitled "Sample composition alters association between age and brain structure." This code was generated using SAS version 9.3 and SUDAAN 11.0 was used to complete all analyses.
