# Supplementary material for: Sample composition alters associations between age and brain structure
Source: Nat Commun. 2017 Oct 12;8:874. doi: 10.1038/s41467-017-00908-7 (PMC5638928; doi:10.1038/s41467-017-00908-7)
Supplement: Supplementary file 4 — Supplementary Data 1 [file 41467_2017_908_MOESM4_ESM.docx]

**Supplementary Data 1**

SAS Statistical Code

Here, we provide the statistical code used to generate the results summarized in this manuscript titled “Sample composition alters associations between age and brain structure.” SAS version 9.3 and SUDAAN 11.0 was used to complete all analyses.

libname pums '/pums';

libname kneuro '/neurok';

************** PUMS data - get census level data;

*household data;

**data** pumsHa;

set pums.psam_husa;

**run**;

**data** pumsHb;

set pums.psam_husb;

**run**;

**data** pumsHc;

set pums.psam_husc;

**run**;

**data** pumsHd;

set pums.psam_husd;

**run**;

**data** pumsHouse;

set pumsHa pumsHb pumsHc pumsHd;

**run**;

*individual level data;

**data** pumsa;

set pums.psam_pusa (keep = RELP RC SFR serialno hisp rac1p SEMP sex sch schg schl AGEP WAGP PWGTP1 PWGTP);

**run**;

**data** pumsb;

set pums.psam_pusb (keep = RELP RC SFR serialno hisp rac1p SEMP sex sch schg schl AGEP WAGP PWGTP1 PWGTP);

**run**;

**data** pumsc;

set pums.psam_pusc (keep = RELP RC SFR serialno hisp rac1p SEMP sex sch schg schl AGEP WAGP PWGTP1 PWGTP);

**run**;

**data** pumsd;

set pums.psam_pusd (keep = RELP RC SFR serialno hisp rac1p SEMP sex sch schg schl AGEP WAGP PWGTP1 PWGTP);

**run**;

*individual combined file;

**Data** PUMS;

set pumsa pumsb pumsc pumsd;

*creating hispanic ethnicity;

hisp2 = hisp ***1**;

if HISP2 >**1** then hispeth =**2**; else hispeth =**1**;

*age group we want to look at. WE have ages 3-18 in PING;

age = agep***1**;

if **3**<=age <**7** then agecat = **1**;

else if **7**<=age <**11** then agecat = **2**;

else if **11**<=age <**15** then agecat = **3**;

else if **15**<=age <**19** then agecat = **4**;

**run**;

**proc** **sort** data = pumsHouse; by serialno; **run**;

**proc** **sort** data = PUMS; by serialno; **run**;

*combining individual and household level files;

**data** combPUM(keep = AGEP HINCP HISP HUPAC NOC NP PUMA

RAC1P hispeth RC REGION SCH SCHG SCHL SEMP SEX

SFR WAGP age agecat houseInc relp serialno IDnew kidof refperson kidSerial kidSerial2

refserial parSerial parentof kidrefper PotentialParSer PotentialPar pweight pWeight1 houseweight

income3cat education2);

merge PUMS pumshouse; by serialno;

*creating new unique ID;

IDNewCh=_n_;

IDnew = IDNewCh***1**;

*steps taken to clarify family. Doing this to get parental education information for the children;

if relp in (**2**,**3**,**4**) then kidOf = **1**; else kidof = **0**; *relp = relationship to reference person. 2/3/4 are children in the household;

if relp = **0** then refPerson = **1**; else refPerson = **0**; *relp =0 is reference person;

if kidof = **1** then kidSerial = serialno;

if refperson = **1** then refSerial = serialno;

*if reference is between 3-18: ;

if agecat ~=**.** and refperson = **1** then kidRefPer = **1**; else kidrefper = **0**;

if relp =**6** and kidrefper = **1** then ParentOf = **1**; else parentof = **0**;

if parentof = **1** then parSerial = serialno;

if kidof = **1** or kidrefper = **1** then kidSerial2 = serialno; *giving child the family's ID;

if parentof =**1** or refperson = **1** then PotentialParSer = serialno; *giving potential parent the family's ID;

if parentof =**1** or refperson = **1** then PotentialPar = **1**; else potentialpar = **0**; *creating variable for a potential parent;

*survey weights;

pWeight = PWGTP***1**;

pWeight1 = PWGTP1***1**;

houseweight = WGTP***1**;

*highest education level each respondent received, 4 groups: High school or less, some college, college degree, more than 4-year college;

if **0**< schl<**18** then education2 = **1**;

else if schl in (**18**,**19**,**20**) then education2 =**2**;

else if schl =**21** then education2 = **3**;

else if **21**<schl<=**24** then education2 = **4**;

*household income variable split into three groups, <40k, 40k-100k, >=100k;

houseinc = HINCP***1**;

if **0**<=houseInc<**40000** then Income3cat =**1**;

else if **40000**<=houseInc<**100000** then income3cat =**2**;

else if houseInc>=**100000** then income3cat =**3**;

**run**;

*use SQL to connect parents to children in same family/household of PUMS;

**proc** **sql**;

create table PumsWithEd AS

select distinct *

from combPUM join (

select distinct serialno as ParSerialno, education2 AS parentEd, idnew AS parIDNEW

from combPUM

where PotentialPar =**1**)

on (kidSerial2 = parserialno);

**quit**;

*calculated the number of people in each home;

**proc** **sql**;

create table NumInHouse AS

select count(serialno), idnew

from combPUM

group by serialno;

**quit**;

*creating variable in dataset that specifies parental/child connection has occured;

**data** PumsWithEd;

set PumsWithEd;

inEd = **1**;

**run**;

**proc** **sort** data = PumsWithEd; by idnew;

**proc** **sort** data = NumInHouse; by idnew;

**data** pumswithed2;

merge PumsWithEd numinhouse; by idnew;

if ined =**.** then delete; *If there is not a household/family in PUMS with a parent-child then it is deleted;

if agecat = **.** then delete;

if hispeth = **2** then HispCorr = **1**; else if hispeth = **1** then hispCorr = **2**;

if rac1p = **1** then cenRace = **1**; *white;

else if rac1p =**2** then cenRace = **2**; *black;

else if hispCorr = **1** then cenRace = **3**; *hispanic;

else if rac1p = **9** then cenRace = **5**; *two or more races;

else if rac1p in (**3**,**4**,**5**,**6**,**7**,**8**) then cenRace = **4**; *other race;

*create income variable with a category for missing on income response;

incomemiss= income3cat;

if income3cat = **.** then incomemiss = **10**;

**run**;

*values for creating a census weight;

**proc** **freq** data = pumswithed2;

weight pweight;

table sex cenrace agecat parented income3cat incomemiss; **run**;

************************** PING DATA **************************;

**data** nuse;

set kneuro.PING_data_AHedits;

*dummy variables;

weight1 = **1**;

nest1 = **1**;

HUH =**1**;

*round age;

Rage = round(age, **1**);

*4 age categories;

if **3**<=rage <**7** then ragecat = **1**;

else if **7**<=rage <**11** then ragecat = **2**;

else if **11**<=rage <**15** then ragecat = **3**;

else if **15**<=rage <**19** then ragecat = **4**;

*race renames;

hispeth = FDH_1_Hispanic_or_Latino***1**;

PacificIslander = FDH_2_Pacific_Islander***1**;

asian = FDH_2_Asian***1**;

black = FDH_2_African_American***1**;

nativeAmerican = FDH_2_American_Indian***1**;

white = FDH_2_White***1**;

raceSum = sum(white, black, nativeamerican, asian, PacificIslander); *2+ races;

*final race variable;

if hispeth = **0** and asian = **0** and black =**0** and nativeAmerican=**0** and PacificIslander = **0** and white = **1** then CenRace = **1**; *white only;

else if hispeth= **0** and asian = **0** and black =**1** and nativeAmerican=**0** and PacificIslander = **0** and white = **0** then cenRace =**2**; *black only;

else if hispeth = **1** then cenRace = **3**; *hispanic;

else if hispeth = **0** and raceSum>=**2** then cenRace = **5**; *more than one race;

else if hispeth = **0** and asian = **1** or pacificIslander = **1** or nativeamerican=**1** then cenrace = **4**; *asian/pacific Islander/native american;

*highest education level parent 1 received, 4 groups: High school or less, some college, college degree, more than 4-year college;

if FDH_Guardian_1_Edu in (**1**,**2**,**3**,**4**) then Par1Ed4cat = **1**;

else if FDH_Guardian_1_Edu in (**5**) then Par1Ed4cat = **2**;

else if FDH_Guardian_1_Edu =**6** then Par1Ed4cat =**3**;

else if FDH_Guardian_1_Edu in (**7**) then Par1Ed4cat =**4**;

*highest education level parent 2 received, 4 groups: High school or less, some college, college degree, more than 4-year college;

if FDH_Guardian_2_Edu in (**1**,**2**,**3**,**4**) then Par2Ed4cat = **1**;

else if FDH_Guardian_2_Edu in(**5**) then Par2Ed4cat = **2**;

else if FDH_Guardian_2_Edu in(**6**) then Par2Ed4cat = **3**;

else if FDH_Guardian_2_Edu in (**7**) then Par2Ed4cat =**4**;

*parhighEd3 = highest parental education between the two;

if Par1Ed4cat>=Par2Ed4cat then parHighEd3 = Par1Ed4cat;

else if Par2Ed4cat > Par1Ed4cat then parHighEd3 = Par2Ed4cat;

*household income for child;

if FDH_3_Household_Income in (**1**,**2**,**3**,**4**,**5**) then income3cat = **1**;

else if FDH_3_Household_Income in (**6**,**7**) then income3cat =**2**;

else if FDH_3_Household_Income>**7** then income3cat =**3**;

*include missing income as a category;

incomemiss = income3cat;

if income3cat = **.** then incomemiss = **10**;

**run**;

*if missing on race or not in ages from 3-18, then delete;

**data** NWuse2;

set nuse;

if ragecat = **.** then delete;

if cenrace = **.** then delete;

if parhighed3 = **.** then delete;

**run**;

*Weighting Code;

**PROC** **WTADJUST** DATA=NWuse2 ADJUST=post NOTSORTED;

WEIGHT weight1;

nest ragecat;

CLASS cenrace gender parhighed3 incomemiss;

MODEL huh=cenrace gender parhighed3 incomemiss;

WTMAX **80000**;

WTMIN **15**;

LOWERBD **15**;

CENTER **42700**;

UPPERBD **80000**;

POSTWGT

/* total */ **58806391**

/* race */ **41100215** **7974668** **4419405** **3225478** **2086625**

/* gender */**30138428** **28667963**

/* parented*/ **21889657** **19173950** **11100192** **6642592**

/* income*/ **19691520** **23832118** **15260552** **22201**

;

IDVAR subjid ragecat;

OUTPUT IDVAR wtfinal/filename=mod_weights5

predicted=all replace;

**run**;

**proc** **sort** data = mod_weights5; by subjid;

**proc** **sort** data = nwuse2; by subjid;

**data** weightedNeuro;

merge mod_weights5 (keep = wtfinal subjid) nwuse2; by subjid;

ageSq = rAge*rAge;

*scanner system/location that was used to scann child;

if Manufacturer = "GE MEDICAL SYSTEMS" and ManufacturersModelName = "DISCOVERY MR750" then scanner5c = **1**;

else if Manufacturer = "GE MEDICAL SYSTEMS" and ManufacturersModelName = "SIGNA HDx" then scanner5c = **2**;

else if Manufacturer = "Philips Medical Systems" and ManufacturersModelName = "Achieva" then scanner5c = **3**;

else if Manufacturer = "SIEMENS" and ManufacturersModelName = "TrioTim" then scanner5c = **4**;

else scanner5c = **5**;

if cenrace =**.** then delete;

if parhighed3 =**.** then delete;

**run**;

*table 1 percentages;

**proc** **freq** data = weightedNeuro;

table cenrace gender ragecat parhighed3 income3cat;

**run**;

**proc** **freq** data = weightedNeuro;

weight wtfinal;

table cenrace gender ragecat parhighed3 income3cat;

**run**;

**proc** **sort** data = weightedNeuro; by subjid;

**proc** **sort** data = kneuro.CC_insula_measures; by subjid;

**proc** **sort** data = kneuro.PING_AddedValues_10_06; by subjid;

*adding in other brain regions;

**data** wneuro;

merge weightedNeuro kneuro.CC_insula_measures kneuro.PING_AddedValues_10_06; by subjid;

if wtfinal = **.** then delete;

**run**;

*get mean of age to center age variables;

**proc** **means** data = wneuro;

var rage;

**run**;

*get weighted mean of age to center age variables;

**proc** **means** data = wneuro;

weight wtfinal;

var rage;

**run**;

*******;

**data** wneuro_safety;

set wneuro;

*control for scanner in models, so only keep those observations with scanner;

if scanner5c = **5** then scanner5c =**.**;

*age category for 2-year age groups;

if rage in (**3**,**4**) then ragecat = **1**;

else if rage in (**5**,**6**) then ragecat = **2**;

else if rage in (**7**,**8**) then ragecat = **3**;

else if rage in (**9**,**10**) then ragecat = **4**;

else if rage in (**11**,**12**) then ragecat = **5**;

else if rage in (**13**,**14**) then ragecat = **6**;

else if rage in (**15**,**16**) then ragecat = **7**;

else if rage in (**17**,**18**) then ragecat = **8**;

*use ragecat for 4-category;

if **3**<=rage <**7** then ragecat2 = **1**;

else if **7**<=rage <**11** then ragecat2 = **2**;

else if **11**<=rage <**15** then ragecat2 = **3**;

else if **15**<=rage <**19** then ragecat2 = **4**;

*Get age, age squared, and age cubed of mean centered values for unweighted values;

meanAge = **10.4475043**;

Age_mean = Rage-meanage;

Age_meansq = age_mean*age_mean;

Age_meancu = age_mean*age_mean*age_mean;

*Get age, age squared, and age cubed of mean centered values for weighted values;

meanAgeW = **10.8460994**;

AgeW_mean = RAge-meanAgeW;

AgeW_meansq = AgeW_mean*AgeW_mean;

AgeW_meancu = AgeW_mean*AgeW_mean*AgeW_mean;

*Frontal Lobe;

*Frontal Surface Area, lefthand side;

frontalSA_LH = sum(MRI_cort_area_ctx_lh_superiorfro,

MRI_cort_area_ctx_lh_rostralmidd,MRI_cort_area_ctx_lh_caudalmiddl,

MRI_cort_area_ctx_lh_parsopercul,MRI_cort_area_ctx_lh_parstriangu,

MRI_cort_area_ctx_lh_parsorbital,MRI_cort_area_ctx_lh_lateralorbi,

MRI_cort_area_ctx_lh_medialorbit,MRI_cort_area_ctx_lh_precentral,

MRI_cort_area_ctx_lh_paracentral,MRI_cort_area_ctx_lh_frontalpole,

MRI_cort_area_ctx_lh_rostralante,MRI_cort_area_ctx_lh_caudalanter);

*Frontal Surface Area, righthand side;

frontalSA_RH = sum(MRI_cort_area_ctx_RH_superiorfro,

MRI_cort_area_ctx_RH_rostralmidd,MRI_cort_area_ctx_RH_caudalmiddl,

MRI_cort_area_ctx_RH_parsopercul,MRI_cort_area_ctx_RH_parstriangu,

MRI_cort_area_ctx_RH_parsorbital,MRI_cort_area_ctx_RH_lateralorbi,

MRI_cort_area_ctx_RH_medialorbit,MRI_cort_area_ctx_RH_precentral,

MRI_cort_area_ctx_RH_paracentral,MRI_cort_area_ctx_RH_frontalpole,

MRI_cort_area_ctx_RH_rostralante,MRI_cort_area_ctx_rh_caudalanter);

*Frontal Thickness, lefthand side;

frontalTH_LH = mean(MRI_cort_thick_ctx_lh_superiorfr,

MRI_cort_thick_ctx_lh_rostralmid,MRI_cort_thick_ctx_lh_caudalmidd,

MRI_cort_thick_ctx_lh_parsopercu,MRI_cort_thick_ctx_lh_parstriang,

MRI_cort_thick_ctx_lh_parsorbita,MRI_cort_thick_ctx_lh_lateralorb,

MRI_cort_thick_ctx_lh_medialorbi,MRI_cort_thick_ctx_lh_precentral,

MRI_cort_thick_ctx_lh_paracentra,MRI_cort_thick_ctx_lh_frontalpol,

MRI_cort_thick_ctx_lh_rostralant,MRI_cort_thick_ctx_lh_caudalante);

*Frontal Thickness, righthand side;

frontalTH_RH = mean(MRI_cort_thick_ctx_RH_superiorfr,

MRI_cort_thick_ctx_RH_rostralmid,MRI_cort_thick_ctx_RH_caudalmidd,

MRI_cort_thick_ctx_RH_parsopercu,MRI_cort_thick_ctx_RH_parstriang,

MRI_cort_thick_ctx_RH_parsorbita,MRI_cort_thick_ctx_RH_lateralorb,

MRI_cort_thick_ctx_RH_medialorbi,MRI_cort_thick_ctx_RH_precentral,

MRI_cort_thick_ctx_RH_paracentra,MRI_cort_thick_ctx_RH_frontalpol,

MRI_cort_thick_ctx_RH_rostralant,MRI_cort_thick_ctx_rh_caudalante);

*Parietal Lobe;

*Parietal Surface Area, lefthand side;

parietalSA_LH = sum(MRI_cort_area_ctx_lh_superiorpar,

MRI_cort_area_ctx_lh_inferiorpar,MRI_cort_area_ctx_lh_supramargin,

MRI_cort_area_ctx_lh_postcentral,MRI_cort_area_ctx_lh_precuneus,

MRI_cort_area_ctx_lh_posteriorci,MRI_cort_area_ctx_lh_isthmuscing);

*Parietal Surface Area, righthand side;

parietalSA_RH = sum(MRI_cort_area_ctx_RH_superiorpar,

MRI_cort_area_ctx_RH_inferiorpar,MRI_cort_area_ctx_RH_supramargin,

MRI_cort_area_ctx_RH_postcentral,MRI_cort_area_ctx_RH_precuneus,

MRI_cort_area_ctx_RH_posteriorci,MRI_cort_area_ctx_RH_isthmuscing);

*Parietal Thickness, lefthand side;

parietalTH_LH = mean(MRI_cort_thick_ctx_lh_superiorpa,

MRI_cort_thick_ctx_lh_inferiorpa,MRI_cort_thick_ctx_lh_supramargi,

MRI_cort_thick_ctx_lh_postcentra,MRI_cort_thick_ctx_lh_precuneus,

MRI_cort_thick_ctx_lh_posteriorc,MRI_cort_thick_ctx_lh_isthmuscin);

*Parietal Thickness, righthand side;

parietalTH_RH = mean(MRI_cort_thick_ctx_RH_superiorpa,

MRI_cort_thick_ctx_RH_inferiorpa,MRI_cort_thick_ctx_RH_supramargi,

MRI_cort_thick_ctx_RH_postcentra,MRI_cort_thick_ctx_RH_precuneus,

MRI_cort_thick_ctx_RH_posteriorc,MRI_cort_thick_ctx_RH_isthmuscin);

*Temporal Lobe;

*Temporal Surface Area, lefthand side;

temporalSA_LH = sum(MRI_cort_area_ctx_lh_superiortem,

MRI_cort_area_ctx_lh_middletempo,MRI_cort_area_ctx_lh_inferiortem,

MRI_cort_area_ctx_lh_bankssts,MRI_cort_area_ctx_lh_fusiform,

MRI_cort_area_ctx_lh_transverset,MRI_cort_area_ctx_lh_entorhinal,

MRI_cort_area_ctx_lh_temporalpol,MRI_cort_area_ctx_lh_parahippoca);

*Temporal Surface Area, righthand side;

temporalSA_RH = sum(MRI_cort_area_ctx_RH_superiortem,

MRI_cort_area_ctx_RH_middletempo,MRI_cort_area_ctx_RH_inferiortem,

MRI_cort_area_ctx_RH_bankssts,MRI_cort_area_ctx_RH_fusiform,

MRI_cort_area_ctx_RH_transverset,MRI_cort_area_ctx_RH_entorhinal,

MRI_cort_area_ctx_RH_temporalpol,MRI_cort_area_ctx_RH_parahippoca);

*Temporal Thickness, lefthand side;

temporalTH_LH = mean(MRI_cort_thick_ctx_lh_superiorte,

MRI_cort_thick_ctx_lh_middletemp,MRI_cort_thick_ctx_lh_inferiorte,

MRI_cort_thick_ctx_lh_bankssts,MRI_cort_thick_ctx_lh_fusiform,

MRI_cort_thick_ctx_lh_transverse,MRI_cort_thick_ctx_lh_entorhinal,

MRI_cort_thick_ctx_lh_temporalpo,MRI_cort_thick_ctx_lh_parahippoc);

*Temporal Thickness, righthand side;

temporalTH_RH = mean(MRI_cort_thick_ctx_RH_superiorte,

MRI_cort_thick_ctx_RH_middletemp,MRI_cort_thick_ctx_RH_inferiorte,

MRI_cort_thick_ctx_RH_bankssts,MRI_cort_thick_ctx_RH_fusiform,

MRI_cort_thick_ctx_RH_transverse,MRI_cort_thick_ctx_RH_entorhinal,

MRI_cort_thick_ctx_RH_temporalpo,MRI_cort_thick_ctx_RH_parahippoc);

*Occipital Lobe;

*Occipital Surface Area, lefthand side;

occipitalSA_LH = sum(MRI_cort_area_ctx_lh_lateralocci,MRI_cort_area_ctx_lh_lingual,

MRI_cort_area_ctx_lh_cuneus,MRI_cort_area_ctx_lh_pericalcari);

*Occipital Surface Area, righthand side;

occipitalSA_RH = sum(MRI_cort_area_ctx_RH_lateralocci,MRI_cort_area_ctx_RH_lingual,

MRI_cort_area_ctx_RH_cuneus,MRI_cort_area_ctx_RH_pericalcari);

*Occipital Thickness, lefthand side;

occipitalTH_LH = sum(MRI_cort_thick_ctx_lh_lateralocc,MRI_cort_thick_ctx_lh_lingual,

MRI_cort_thick_ctx_lh_cuneus,MRI_cort_thick_ctx_lh_pericalcar);

*Occipital Thickness, rightthand side;

occipitalTH_RH = sum(MRI_cort_thick_ctx_RH_lateralocc,MRI_cort_thick_ctx_RH_lingual,

MRI_cort_thick_ctx_RH_cuneus,MRI_cort_thick_ctx_RH_pericalcar);

***** sum for subcortal total volume;

*Left Subcortal Volume;

leftSubcort_vol= sum(MRI_subcort_vol_Left_Thalamus_Pr, MRI_subcort_vol_Left_Caudate, MRI_subcort_vol_Left_Putamen,

MRI_subcort_vol_Left_Pallidum, MRI_subcort_vol_Left_Hippocampus, MRI_subcort_vol_Left_Amygdala, MRI_subcort_vol_Left_Accumbens_a);

*Right Subcortal Volume;

rightSubcort_vol= sum(MRI_subcort_vol_Right_Thalamus_P, MRI_subcort_vol_Right_Caudate, MRI_subcort_vol_Right_Putamen,

MRI_subcort_vol_Right_Pallidum, MRI_subcort_vol_Right_Hippocampu, MRI_subcort_vol_Right_Amygdala, MRI_subcort_vol_Right_Accumbens_);

*Total Subcortal Volume;

totalSubCort_vol =sum(leftSubcort_vol,rightSubcort_vol);

*Hippocampus (bilateral) Volume;

totalHippocam_vol = sum(MRI_subcort_vol_Left_Hippocampus, MRI_subcort_vol_Right_Hippocampu);

*Amygdala (bilateral) Volume;

totalAmygdala_vol = sum(MRI_subcort_vol_Left_Amygdala, MRI_subcort_vol_Right_Amygdala);

*Basal Ganglia (bilateral) Volume;

totalBasalGang_vol = sum(MRI_subcort_vol_Left_Caudate, MRI_subcort_vol_Right_Caudate,

MRI_subcort_vol_Left_Putamen,MRI_subcort_vol_Right_Putamen,MRI_subcort_vol_Left_Pallidum,

MRI_subcort_vol_Right_Pallidum, MRI_subcort_vol_Left_Accumbens_a, MRI_subcort_vol_Right_Accumbens_);

**run**;

*This macro outputs the linear, quadratic, and cubic unweighted and weighted models

for thickness and surface area brain regions;

**%macro** part1run_Safe(inputb, title);

*Unweighted linear;

proc genmod data = wneuro_safety;

model &inputB = Age_mean ;

title &inputB;

run;

*weighted linear;

proc genmod data = wneuro_safety;

weight wtfinal;

model &inputB = Agew_mean ;

title &inputB;

run;

*Unweighted quadratic;

proc genmod data = wneuro_safety;

model &inputB = Age_mean Age_meansq ;

title &inputB;

run;

*Weighted quadratic;

proc genmod data = wneuro_safety;

weight wtfinal;

model &inputB = Agew_mean Agew_meansq ;

title &inputB;

run;

*Unweighted cubic;

proc genmod data = wneuro_safety;

model &inputB = Age_mean Age_meansq Age_meancu ;

title &inputB;

run;

*Weighted cubic;

proc genmod data = wneuro_safety;

weight wtfinal;

model &inputB = Agew_mean Agew_meansq Agew_meancu ;

title &inputB;

run;

**%mend**;

*Thickness measures;

%***part1run_safe***(MRI_cort_thick_ctx_mean, Thickness: Total Mean);

%***part1run_safe***(MRI_cort_thick_ctx_lh_mean, Thickness: LH Mean);

%***part1run_safe***(MRI_cort_thick_ctx_rh_mean, Thickness: RH Mean);

%***part1run_safe***(occipitalTH_LH, TH: occipital LH);

%***part1run_safe***(occipitalTH_RH, TH: occipital RH);

%***part1run_safe***(temporalTH_LH, TH: temporal LH);

%***part1run_safe***(temporalTH_RH, TH: temporal RH);

%***part1run_safe***(parietalTH_LH, TH: parietal LH);

%***part1run_safe***(parietalTH_RH, TH: parietal RH);

%***part1run_safe***(frontalTH_LH, TH: frontal LH);

%***part1run_safe***(frontalTH_RH, TH: frontal RH);

*Surface Area measures;

%***part1run_safe***(MRI_cort_area_ctx_total, Area: Total);

%***part1run_safe***(MRI_cort_area_ctx_lh_total, Area: LH Total);

%***part1run_safe***(MRI_cort_area_ctx_rh_total, Area: RH Total);

%***part1run_safe***(occipitalSA_LH, SA: occipital LH);

%***part1run_safe***(occipitalSA_RH, SA: occipital RH);

%***part1run_safe***(temporalSA_LH, SA: temporal LH);

%***part1run_safe***(temporalSA_rH, SA: temporal RH);

%***part1run_safe***(parietalSA_LH, SA: parietal LH);

%***part1run_safe***(parietalSA_rH, SA: parietal RH);

%***part1run_safe***(frontalSA_LH, SA: frontal LH);

%***part1run_safe***(frontalSA_RH, SA: frontal RH);

*Macro graphs thickness and area weighted and unweighted models. Takes input of

unweighted (inp1) and weighted (inp2) best fitting models from macro above (linear, quadratic, cubic).

Genmod analyses output predicted values by age. Then unweighted and weighted curves are graphed by age.

Predicted values for unweighted models = opUW

Predicted values for weighted models = opW

;

**%macro** graphItSafe(inputb, side, title, inp1, inp2);

*Unweighted model;

%if %upcase(&inp1)=LIN %then %do;

proc genmod data = wneuro_safety ;

model &inputB = Age_mean ;

output out = opUWdata (keep = wtfinal opUW rage Age_mean subjID) PREDICTED = opUW;

title &inputB;

run;

%end;

%else %if %upcase(&inp1)=QUAD %then %do;

proc genmod data = wneuro_safety ;

model &inputB = Age_mean Age_meansq ;

output out = opUWdata (keep = wtfinal opUW rage Age_mean subjID) PREDICTED = opUW;

title &inputB;

run;

%end;

%else %if %upcase(&inp1)=CUB %then %do;

proc genmod data = wneuro_safety ;

model &inputB = Age_mean Age_meansq Age_meancu ;

output out = opUWdata (keep = wtfinal opUW rage Age_mean subjID) PREDICTED = opUW;

title &inputB;

run;

%end;

*Weighted model;

%if %upcase(&inp2)=LIN %then %do;

proc genmod data = wneuro_safety ;

weight wtfinal;

model &inputB = Agew_mean ;

output out = opWdata (keep = wtfinal opW rage Age_mean subjID) PREDICTED = opW;

title &inputB;

run;

%end;

%else %if %upcase(&inp2)=QUAD %then %do;

proc genmod data = wneuro_safety ;

weight wtfinal;

model &inputB = Agew_mean Agew_meansq ;

output out = opWdata (keep = wtfinal opW rage Agew_mean subjID) PREDICTED = opW;

title &inputB;

run;

%end;

%else %if %upcase(&inp2)=CUB %then %do;

proc genmod data = wneuro_safety ;

weight wtfinal;

model &inputB = Agew_mean Agew_meansq Agew_meancu ;

output out = opWdata (keep = wtfinal opW rage Agew_mean subjID) PREDICTED = opW;

title &inputB;

run;

%end;

proc sort data = opUWdata; by subjid;

proc sort data = opWdata; by subjid;

*File of unweighted and weighted predicted values;

data ranTogether;

merge opUWdata opWdata; by subjid;

run;

*Get mean value of predicted models by age (3-18);

proc sort data = ranTogether; by rage;

proc means data=ranTogether noprint;

by rage;

var opUW opW ;

output out=testMeanA mean=;

run;

%if %upcase(&inp1)=LIN %then %do;

symbol1 font=marker value=U

color=blue

interpol=join

height=**1.5** width =**2**;

%end;

%else %if %upcase(&inp1)=QUAD %then %do;

symbol1 font=marker value=C

color=blue

interpol=join

height=**1.5** width =**2**;

%end;

%else %if %upcase(&inp1)=CUB %then %do;

symbol1 font=marker value=P

color=blue

interpol=join

height=**1.5** width =**2**;

%end;

%if %upcase(&inp2)=LIN %then %do;

symbol2 font=marker value=U

color=red

interpol=join

height=**1.5** width =**2**;

%end;

%else %if %upcase(&inp2)=QUAD %then %do;

symbol2 font=marker value=C

color=red

line=**2**

interpol=join

height=**1.5** width =**2**;

%end;

%else %if %upcase(&inp2)=CUB %then %do;

symbol2 font=marker value=P

color=red

line=**2**

interpol=join

height=**1.5** width =**2**;

%end;

legend1 value=(

%if %upcase(&inp1)=LIN %then %do;

tick=**1** justify=c "Unweighted Linear"

%end;

%else %if %upcase(&inp1)=QUAD %then %do;

tick=**1** justify=c "Unweighted Quadratic"

%end;

%else %if %upcase(&inp1)=CUB %then %do;

tick=**1** justify=c "Unweighted Cubic"

%end;

%if %upcase(&inp2)=LIN %then %do;

tick=**2** justify=c "Weighted Linear"

%end;

%else %if %upcase(&inp2)=QUAD %then %do;

tick=**2** justify=c "Weighted Quadratic"

%end;

%else %if %upcase(&inp2)=CUB %then %do;

tick=**2** justify=c "Weighted Cubic"

%end;

)

shape=symbol(**8**,**1**)

position=(bottom center inside)

mode=share

label=none frame;

axis3 order=(**3** to **18** by **3**) minor=(n=**2**) label=( 'Age (year)' font='Helvetica/Bold' height=**1.75**);

axis2 major=(n=**5**) minor=(n=**3**) label=(a=**90** j=c "&side" font='Helvetica/Bold' height=**1.75**);

filename outfile "/Area &title..tiff";

goptions device=gif gsfname=outfile gsfmode=replace ftext='Helvetica/Bold' htext=**14**pt;

proc sort data = testMeanA; by rage; run;

proc gplot data=testMeanA;

plot opuW*rage opW*rage/overlay legend = legend1 haxis=axis3 vaxis = axis2;

title "&title";

run;

quit;

**%mend**;

%***graphItSafe***(MRI_cort_thick_ctx_lh_mean, Thickness (mm), LH Mean Thickness, cub, lin);

%***graphItSafe***(MRI_cort_thick_ctx_rh_mean, Thickness (mm), RH Mean Thickness, cub, cub);

%***graphItSafe***(MRI_cort_thick_ctx_mean, Thickness (mm), Total Mean Thickness, cub, cub);

%***graphItSafe***(occipitalTH_LH,Thickness (mm), LH Occipital Region,cub, quad);

%***graphItSafe***(occipitalTH_RH,Thickness (mm), RH Occipital Region,cub, quad);

%***graphItSafe***(temporalTH_LH,Thickness (mm), LH Temporal Region,lin,lin);

%***graphItSafe***(temporalTH_rH,Thickness (mm), RH Temporal Region,lin, lin);

%***graphItSafe***(parietalTH_LH,Thickness (mm), LH Parietal Region,lin, lin);

%***graphItSafe***(parietalTH_RH,Thickness (mm), RH Parietal Region,quad, cub);

%***graphItSafe***(frontalTH_LH, Thickness (mm), LH Frontal Region,cub, cub);

%***graphItSafe***(frontalTH_RH, Thickness (mm), RH Frontal Region,quad, quad);

%***graphItSafe***(MRI_cort_area_ctx_total, Surface Area (mm2), Total Cortical Surface Area, quad, cub);

%***graphItSafe***(MRI_cort_area_ctx_lh_total, Surface Area (mm2), LH Cortical Surface Area, quad, cub);

%***graphItSafe***(MRI_cort_area_ctx_rh_total, Surface Area (mm2), RH Cortical Surface Area, quad, cub);

%***graphItSafe***(occipitalSA_LH,Surface Area (mm2), LH Occipital Region,quad, cub);

%***graphItSafe***(occipitalSA_RH,Surface Ahrea (mm2), RH Occipital Region,quad, cub);

%***graphItSafe***(temporalSA_LH,Surface Area (mm2), LH Temporal Region,quad, cub);

%***graphItSafe***(temporalSA_rH,Surface Area (mm2), RH Temporal Region,quad, cub);

%***graphItSafe***(parietalSA_LH,Surface Area (mm2), LH Parietal Region,cub, cub);

%***graphItSafe***(parietalSA_RH,Surface Area (mm2), RH Parietal Region, cub,cub);

%***graphItSafe***(frontalSA_LH, Surface Area (mm2), LH Frontal Region,quad, cub);

%***graphItSafe***(frontalSA_RH, Surface Area (mm2), RH Frontal Region,quad, cub);

%***graphItSafe***(MRI_subcort_vol_IntracranialVolu, Volume (mm3), Subcortical Intracranial Volume, quad, quad);

*This macro outputs the linear, quadratic, and cubic unweighted and weighted models

for volume brain regions. Cotrolling for intracranial volume;

**%macro** part1runv(inputb, title);

*Unweighted linear;

proc genmod data = wneuro_safety;

model &inputB = Age_mean MRI_subcort_vol_IntracranialVolu;

title &inputB;

run;

*Weighted linear;

proc genmod data = wneuro_safety;

weight wtfinal;

model &inputB = Agew_mean MRI_subcort_vol_IntracranialVolu;

title &inputB;

run;

*Unweighted quadratic;

proc genmod data = wneuro_safety;

model &inputB = Age_mean Age_meansq MRI_subcort_vol_IntracranialVolu;

title &inputB;

run;

*Weighted quadratic;

proc genmod data = wneuro_safety;

weight wtfinal;

model &inputB = Agew_mean Agew_meansq MRI_subcort_vol_IntracranialVolu;

title &inputB;

run;

*Unweighted cubic;

proc genmod data = wneuro_safety;

model &inputB = Age_mean Age_meansq Age_meancu MRI_subcort_vol_IntracranialVolu;

title &inputB;

run;

*Weighted cubic;

proc genmod data = wneuro_safety;

weight wtfinal;

model &inputB = Agew_mean Agew_meansq Agew_meancu MRI_subcort_vol_IntracranialVolu;

title &inputB;

run;

**%mend**;

%***part1runV***(MRI_cort_vol_ctx_lh_total, VOL: total LH);

%***part1runV***(MRI_cort_vol_ctx_rh_total, VOL: total RH);

%***part1runV***(MRI_cort_vol_ctx_total, Vol: TOTAL);

%***part1runV***(totalHippocam_vol, VOL: total Hippocampus);

%***part1runV***(totalAmygdala_vol, VOL: total Amygdala);

%***part1runV***(totalBasalGang_vol, VOL: total Basal Ganglia);

%***part1runV***(totalSubCort_vol, VOL: subcortvol);

********** for graphing Volume measures **********;

* Create mean of intracranial volume by two-year age groups to graph the volume

controlling for intracranial volume. Create mean measure for unweighted and weighted models;

**proc** **sort** data = wneuro_safety; by ragecat; **run**;

**proc** **means** data = wneuro_safety noprint;

by ragecat;

var MRI_subcort_vol_IntracranialVolu;

output out=mIntraVol_1162 mean=mIntraVol_1162;

**run**;

**proc** **sort** data = wneuro_safety; by ragecat; **run**;

**proc** **means** data = wneuro_safety noprint;

weight wtfinal;

by ragecat;

var MRI_subcort_vol_IntracranialVolu;

output out=mIntraVol_1162w mean=mIntraVol_1162w;

**run**;

**proc** **sort** data = wneuro_safety; by ragecat; **run**;

**proc** **sort** data = mIntraVol_1162; by ragecat; **run**;

**proc** **sort** data = mIntraVol_1162w; by ragecat; **run**;

* combined data with categorical intracranial volume;

**data** wneuro_safety2 (drop =mIntraVol) ;

merge wneuro_safety mIntraVol_1162 mIntraVol_1162w; by ragecat;

**run**;

**proc** **freq** data = wneuro_safety2;

tables ragecat*mIntraVol_1162*mIntraVol_1162w/list;

ods output list=freq_categ;

**run**;

*calculate the percentage of age categorical var;

**proc** **sql**;

create table percent_categ as

select *, frequency/sum(frequency) as percentNew

from freq_categ

;

**quit**;

* Merge with frequency table to get percent of each age category;

**proc** **sort** data = freq_categ; by ragecat;

**proc** **sort** data = wneuro_safety2; by ragecat;

**data** wneuro_safety3;

merge freq_categ wneuro_safety2; by ragecat;

*rename variables;

percentUse = percent;

mIntraVol_fix = mIntraVol_1162;

mIntraVolW_fix = mIntraVol_1162w;

**run**;

*rename so can merge into output from genmod equation in graphing macro;

**data** percent_categ2 (keep = level1 percentuse);

set percent_categ;

level1 = F_mIntraVol_1162 ***1**;

rename percentNew = percentuse;

**run**;

*rename so can merge with predicted values from genmod equation in graphing macro;

**data** percent_categ2w (keep = level1 percentuse);

set percent_categ;

level1 = F_mIntraVol_1162w ***1**;

rename percentNew = percentuse;

**run**;

*create data 'ageuse' with age and categorical intracranial volume measure to later merge into predicted values from genmod equation in graphing macro (Unweighted);

**proc** **freq** data =wneuro_safety3;

tables mIntraVol_fix*mIntraVolW_fix*rage*age_mean/list;

ods output list=ageuse (keep = rage Age_mean mIntraVol_fix);

**run**;

*create data 'ageuseW' with age and categorical intracranial volume measure to later merge into predicted values from genmod equation in graphing macro (weighted);

**proc** **freq** data =wneuro_safety3;

tables mIntraVol_fix*mIntraVolW_fix*rage*agew_mean/list;

ods output list=ageuseW (keep = rage Agew_mean mIntraVolw_fix);

**run**;

*Macro to graph volume regions. macro inputs 'inp1' and 'inp2' determine if the

equation for unweighted and weighted respectively should be linear, quadratic, or cubic;

**%macro** graphItvSafe(inputb, side, title, inp1, inp2);

%if %upcase(&inp1)=LIN %then %do;

*UWests saves the output estimates to graph volume of brain regions controlling for categorical intracranial volume (unweighted);

ods output ParameterEstimates =UWests (keep = parameter estimate level1);

proc genmod data = wneuro_safety3 ;

class mIntraVol_fix;

model &inputB = Age_mean mIntraVol_fix;* controling for categorical intracranial volume;

output out = opUWdata (keep = wtfinal opUW rage Age_mean subjID mIntraVol_fix percentUse) PREDICTED = opUW;

title &inputB;

run;

*combine age with parameter estimate output;

proc sql;

create table testx as

select *

from ageuse, uwests

;

quit;

*change level1 to a numerical value;

data testx;

set testx;

level1_2 = level1***1**;

drop level1;

rename level1_2 = level1;

run;

proc sort data = testx; by level1;

proc sort data = percent_categ2; by level1;

*This dataset sets up the values of the brain region as a function of mean centered age;

data uwests2;

merge testx percent_categ2; by level1;

*The variable 'percentUse' is a multiplier for each beta estimate value and

holds the values of the independent variable (mean centered age) to multiply each parameter estimate;

if parameter = "Scale" then delete;

if parameter = "Intercept" then percentuse = **1**;

if parameter = "Age_mean" then percentuse = age_mean;

else if parameter = "mIntraVol_fix" then percentuse =percentuse;

else if percentuse = **.** then percentuse = **1**;

prodCol = percentuse*estimate; *value for each piece of the genmod equation;

run;

*uwestsP creates predV. PredV = total predicted value of volume by each age;

proc sql;

create table uwestsP as

select distinct sum(prodcol) as predV, rage

from uwests2

group by rage

;

quit;

proc sort data = uwests2; by rage; run;

%end;

%else %if %upcase(&inp1)=QUAD %then %do;

*UWests saves the output estimates to graph volume of brain regions controlling for categorical intracranial volume (unweighted);

ods output ParameterEstimates =UWests (keep = parameter estimate level1 );

proc genmod data = wneuro_safety3 ;

class mIntraVol_fix ;

model &inputB = Age_mean Age_meansq mIntraVol_fix;*cenrace gender scanner5c;

output out = opUWdata (keep = wtfinal opUW rage Age_mean subjID mIntraVol_fix percentUse) PREDICTED = opUW;

title &inputB;

*combine age with parameter estimate output;

proc sql;

create table testx as

select *

from ageuse, uwests

;

quit;

*change 'level1' to a numerical variable;

data testx;

set testx;

level1_2 = level1***1**;

drop level1;

rename level1_2 = level1;

run;

proc sort data = testx; by level1;

proc sort data = percent_categ2; by level1;

*This dataset sets up the values of the brain region as a function of mean centered age;

data uwests2;

merge testx percent_categ2; by level1;

*The variable 'percentUse' is a multiplier for each beta estimate value and

holds the values of the independent variable (mean centered age) to multiply each paramter estimate;

if parameter = "Scale" then delete;

if parameter = "Intercept" then percentuse = **1**;

if parameter = "Age_mean" then percentuse = age_mean;

else if parameter = "Age_meansq" then percentuse = age_mean*age_mean;

else if parameter = "mIntraVol_fix" then percentuse =percentuse;

else if percentuse = **.** then percentuse = **1**;

prodCol = percentuse*estimate;

run;

*uwestsP creates predV. PredV = total predicted value of volume by each age;

proc sql;

create table uwestsP as

select distinct sum(prodcol) as predV, rage, age_mean

from uwests2

group by rage

;

quit;

proc sort data = uwests2; by rage; run;

%end;

%else %if %upcase(&inp1)=CUB %then %do;

*UWests saves the output estimates to graph volume of brain regions controlling for categorical intracranial volume (unweighted);

ods output ParameterEstimates =UWests (keep = parameter estimate level1 );

proc genmod data = wneuro_safety3 ;

class mIntraVol_fix ;

model &inputB = Age_mean Age_meansq Age_meancu mIntraVol_fix;*cenrace gender scanner5c;

output out = opUWdata (keep = wtfinal opUW rage Age_mean subjID mIntraVol_fix percentUse) PREDICTED = opUW;

title &inputB;run;

*combine age with parameter estimate output;

proc sql;

create table testx as

select *

from ageuse, uwests;

quit;

*change 'level1' to a numerical variable;

data testx;

set testx;

level1_2 = level1***1**;

drop level1;

rename level1_2 = level1;

run;

proc sort data = testx; by level1;

proc sort data = percent_categ2; by level1;

*This dataset sets up the values of the brain region as a function of mean centered age;

data uwests2;

merge testx percent_categ2; by level1;

*The variable 'percentUse' is a multiplier for each beta estimate value and

holds the values of the independent variable (mean centered age) to multiply each parameter estimate;

if parameter = "Scale" then delete;

if parameter = "Intercept" then percentuse = **1**;

if parameter = "Age_mean" then percentuse = age_mean;

else if parameter = "Age_meansq" then percentuse = age_mean*age_mean;

else if parameter = "Age_meancu" then percentuse = age_mean*age_mean*age_mean;

else if parameter = "mIntraVol_fix" then percentuse =percentuse;

else if percentuse = **.** then percentuse = **1**;

prodCol = percentuse*estimate;

run;

*uwestsP creates predV. PredV = total predicted value of volume by each age;

proc sql;

create table uwestsP as

select distinct sum(prodcol) as predV, rage

from uwests2

group by rage

;

quit;

proc sort data = uwests2; by rage; run;

%end;

%if %upcase(&inp2)=LIN %then %do;

*Wests saves the output estimates to graph volume of brain regions controlling for categorical intracranial volume (weighted);

ods output ParameterEstimates =Wests (keep = parameter estimate level1);

proc genmod data = wneuro_safety3 ;

weight wtfinal;

class mIntraVolw_fix;

model &inputB = Agew_mean mIntraVolw_fix;*cenrace gender scanner5c;

output out = opWdata (keep = wtfinal opW rage Agew_mean subjID mIntraVolw_fix percentUse) PREDICTED = opW;

title &inputB;

run;

*combine age with parameter estimate output;

proc sql;

create table testx2 as

select *

from ageuseW, Wests;

quit;

*change 'level1' to a numerical variable;

data testx2;

set testx2;

level1_2 = level1***1**;

drop level1;

rename level1_2 = level1;

run;

proc sort data = testx2; by level1;

proc sort data = percent_categ2w; by level1;

*This dataset sets up the values of the brain region as a function of mean centered age;

data Wests2;

merge testx2 percent_categ2w; by level1;

*The variable 'percentUse' is a multiplier for each beta estimate value and

holds the values of the independent variable (mean centered age) to multiply each paramter estimate;

if parameter = "Scale" then delete;

if parameter = "Intercept" then percentuse = **1**;

if parameter = "AgeW_mean" then percentuse = Agew_mean;

else if parameter = "mIntraVolW_fix" then percentuse =percentuse;

else if percentuse = **.** then percentuse = **1**;

prodCol = percentuse*estimate;

run;

*westsP creates predV. PredV = total predicted value of volume by each age;

proc sql;

create table westsP as

select distinct sum(prodcol) as predV2, rage

from wests2

group by rage;

quit;

%end;

%else %if %upcase(&inp2)=QUAD %then %do;

*Wests saves the output estimates to graph volume of brain regions controlling for categorical intracranial volume (weighted);

ods output ParameterEstimates =Wests (keep = parameter estimate level1);

proc genmod data = wneuro_safety3 ;

weight wtfinal;

class mIntraVolw_fix;

model &inputB = Agew_mean Agew_meansq mIntraVolw_fix;*cenrace gender scanner5c;

output out = opWdata (keep = wtfinal opW rage Agew_mean subjID mIntraVolw_fix percentUse) PREDICTED = opW;

title &inputB;

run;

*combine age with parameter estimate output;

proc sql;

create table testx2 as

select *

from ageuseW, Wests;

quit;

*change 'level1' to a numerical variable;

data testx2;

set testx2;

level1_2 = level1***1**;

drop level1;

rename level1_2 = level1;

run;

proc sort data = testx2; by level1;

proc sort data = percent_categ2w; by level1;

*This dataset sets up the values of the brain region as a function of mean centered age;

data Wests2;

merge testx2 percent_categ2w; by level1;

*The variable 'percentUse' is a multiplier for each beta estimate value and

holds the values of the independent variable (mean centered age) to multiply each paramter estimate;

if parameter = "Scale" then delete;

if parameter = "Intercept" then percentuse = **1**;

if parameter = "AgeW_mean" then percentuse = Agew_mean;

else if parameter = "AgeW_meansq" then percentuse = Agew_mean*Agew_mean;

else if parameter = "mIntraVolW_fix" then percentuse =percentuse;

else if percentuse = **.** then percentuse = **1**;

prodCol = percentuse*estimate;

run;

*westsP creates predV. PredV = total predicted value of volume by each age;

proc sql;

create table westsP as

select distinct sum(prodcol) as predV2, rage

from wests2

group by rage;

quit;

%end;

%else %if %upcase(&inp2)=CUB %then %do;

*Wests saves the output estimates to graph volume of brain regions controlling for categorical intracranial volume (weighted);

ods output ParameterEstimates =Wests (keep = parameter estimate level1);

proc genmod data = wneuro_safety3 ;

weight wtfinal;

class mIntraVolw_fix;

model &inputB = Agew_mean Agew_meansq Agew_meancu mIntraVolw_fix;* cenrace gender scanner5c;

output out = opWdata (keep = wtfinal opW rage Agew_mean subjID mIntraVolw_fix percentUse) PREDICTED = opW;

title &inputB;

run;

*combine age with parameter estimate output;

proc sql;

create table testx2 as

select *

from ageuseW, Wests;

quit;

*change 'level1' to a numerical variable;

data testx2;

set testx2;

level1_2 = level1***1**;

drop level1;

rename level1_2 = level1;

run;

proc sort data = testx2; by level1;

proc sort data = percent_categ2w; by level1;

*This dataset sets up the values of the brain region as a function of mean centered age;

data Wests2;

merge testx2 percent_categ2w; by level1;

*The variable 'percentUse' is a multiplier for each beta estimate value and

holds the values of the independent variable (mean centered age) to multiply each paramter estimate;

if parameter = "Scale" then delete;

if parameter = "Intercept" then percentuse = **1**;

if parameter = "AgeW_mean" then percentuse = Agew_mean;

else if parameter = "AgeW_meansq" then percentuse = Agew_mean*Agew_mean;

else if parameter = "AgeW_meancu" then percentuse = Agew_mean*Agew_mean*Agew_mean;

else if parameter = "mIntraVolW_fix" then percentuse =percentuse;

else if percentuse = **.** then percentuse = **1**;

prodCol = percentuse*estimate;

run;

*westsP creates predV. PredV = total predicted value of volume by each age;

proc sql;

create table westsP as

select distinct sum(prodcol) as predV2, rage, Agew_mean

from wests2

group by rage

;

quit;

%end;

******** Combine unweighted and weighted curves ************;

proc sort data = westsp; by rage;

proc sort data = Uwestsp; by rage;

data ranTogether;

merge westsp Uwestsp; by rage;

run;

%if %upcase(&inp1)=LIN %then %do;

symbol1 font=marker value=U

color=blue

interpol=join

height=**1.5** width =**2**;

%end;

%else %if %upcase(&inp1)=QUAD %then %do;

symbol1 font=marker value=C

color=blue

interpol=join

height=**1.5** width =**2**;

%end;

%else %if %upcase(&inp1)=CUB %then %do;

symbol1 font=marker value=P

color=blue

interpol=join

height=**1.5** width =**2**;

%end;

%if %upcase(&inp2)=LIN %then %do;

symbol2 font=marker value=U

color=red

interpol=join

height=**1.5** width =**2**;

%end;

%else %if %upcase(&inp2)=QUAD %then %do;

symbol2 font=marker value=C

color=red

line=**2**

interpol=join

height=**1.5** width =**2**;

%end;

%else %if %upcase(&inp2)=CUB %then %do;

symbol2 font=marker value=P

color=red

line=**2**

interpol=join

height=**1.5** width =**2**;

%end;

legend1 value=(

%if %upcase(&inp1)=LIN %then %do;

tick=**1** justify=c "Unweighted Linear"

%end;

%else %if %upcase(&inp1)=QUAD %then %do;

tick=**1** justify=c "Unweighted Quadratic"

%end;

%else %if %upcase(&inp1)=CUB %then %do;

tick=**1** justify=c "Unweighted Cubic"

%end;

%if %upcase(&inp2)=LIN %then %do;

tick=**2** justify=c "Weighted Linear"

%end;

%else %if %upcase(&inp2)=QUAD %then %do;

tick=**2** justify=c "Weighted Quadratic"

%end;

%else %if %upcase(&inp2)=CUB %then %do;

tick=**2** justify=c "Weighted Cubic"

%end;

)

shape=symbol(**8**,**1**)

position=(bottom center inside)

mode=share

label=none frame;

axis3 order=(**3** to **18** by **3**) minor=(n=**2**) label=( 'Age (year)' font='Helvetica/Bold' height=**1.75**);

axis2 major=(n=**5**) minor=(n=**3**) label=(a=**90** j=c "&side" font='Helvetica/Bold' height=**1.75**);

filename outfile "/Vol &title..tiff";

goptions device=gif gsfname=outfile gsfmode=replace;

proc sort data = rantogether; by rage; run;

proc gplot data=rantogether;

plot predv*rage predv2*rage/overlay legend = legend1 haxis=axis3 vaxis = axis2;

title "&title";

run;

quit;

**%mend**;

%***graphItvSafe***(totalHippocam_vol, Volume (mm3), Total Hippocampus, quad, quad);

%***graphItvSafe***(totalAmygdala_vol, Volume (mm3), Total Amygdala, quad, cub);

%***graphItvSafe***(totalBasalGang_vol, Volume (mm3), Total Basal Ganglia, quad, cub);

%***graphItvSafe***(totalSubCort_vol, Volume (mm3), Total Subcortical Volume, quad, cub);

%***graphItvSafe***(MRI_cort_vol_ctx_lh_total, Volume (mm3), LH Cortical Volume, quad, cub);

%***graphItvSafe***(MRI_cort_vol_ctx_rh_total, Volume (mm3), RH Cortical Volume, quad, cub);

%***graphItvSafe***(MRI_cort_vol_ctx_total, Volume (mm3), Total Cortical Volume, quad, cub);
